# Nanoengineering of NiO/MnO_2_/GO Ternary Composite for Use in High-Energy Storage Asymmetric Supercapacitor and Oxygen Evolution Reaction (OER)

**DOI:** 10.3390/nano13010099

**Published:** 2022-12-25

**Authors:** Natasha Arshad, Muhammad Usman, Muhammad Adnan, Muhammad Tayyab Ahsan, Mah Rukh Rehman, Sofia Javed, Zeeshan Ali, Muhammad Aftab Akram, George P. Demopoulos, Asif Mahmood

**Affiliations:** 1 Department of Physics, Government College Women University, Sialkot 51310, Pakistan; 2 School of Chemical and Materials Engineering, National University of Sciences and Technology (NUST), Sector H-12, Islamabad 44000, Pakistan; 3 School of Materials Science and Engineering, Peking University, Beijing 100871, China; 4 Department of Materials Science & Engineering, Pak-Austria Fachhochschule, Institute of Applied Sciences & Technology, Khanpur Road, Mang, Haripur 22650, Pakistan; 5 Materials Engineering, McGill University, Montreal, QC H3A 0C5, Canada; 6 Center for Clean Energy Technology, School of Mathematical and Physical Science, Faculty of Science, University of Technology Sydney, Sydney 2007, Australia; 7 School of Chemical and Biomoecular Engineering, Faculty of Engineering, The University of Sydney, Sydney 2006, Australia

**Keywords:** electrochemical, energy, graphene oxide, MnO_2_, NiO, supercapacitor, nanocomposite electrode

## Abstract

Designing multifunctional nanomaterials for high performing electrochemical energy conversion and storage devices has been very challenging. A number of strategies have been reported to introduce multifunctionality in electrode/catalyst materials including alloying, doping, nanostructuring, compositing, etc. Here, we report the fabrication of a reduced graphene oxide (rGO)-based ternary composite NiO/MnO_2_/rGO (NMGO) having a range of active sites for enhanced electrochemical activity. The resultant sandwich structure consisted of a mesoporous backbone with NiO and MnO_2_ nanoparticles encapsulated between successive rGO layers, having different active sites in the form of Ni-, Mn-, and C-based species. The modified structure exhibited high conductivity owing to the presence of rGO, excellent charge storage capacity of 402 F·g^−1^ at a current density of 1 A·g^−1^, and stability with a capacitance retention of ~93% after 14,000 cycles. Moreover, the NMGO//MWCNT asymmetric device, assembled with NMGO and multi-wall carbon nanotubes (MWCNTs) as positive and negative electrodes, respectively, exhibited good energy density (28 Wh·kg^−1^), excellent power density (750 W·kg^−1^), and capacitance retention (88%) after 6000 cycles. To evaluate the multifunctionality of the modified nanostructure, the NMGO was also tested for its oxygen evolution reaction (OER) activity. The NMGO delivered a current density of 10 mA·cm^−2^ at the potential of 1.59 V versus RHE. These results clearly demonstrate high activity of the modified electrode with strong future potential.

## 1. Introduction

Availability of clean and affordable energy constitutes one of the biggest challenges of today’s world. There have been large efforts devoted to development of new methods and devices to fill the deficit of energy. Increased population and industrial growth are causing high energy demand, which result in the depletion of fossil fuels and many environmental issues. To reduce dependency on fossil fuels, development in renewable energy sources is intensively pursued. At the same time, energy storage is important to obtain maximum energy from renewable energy sources. In this context, batteries, fuel cells, and supercapacitors have become the centre of research and development worldwide. Among these energy storage devices, supercapacitors have become a remarkable candidate in applications demanding high power density. Supercapacitors store energy using electrolytes between their electrodes, which are separated by a semipermeable membrane. This membrane, owing to the pores, allows the ions to pass during charging and discharging. There is still a vacuum in which to develop supercapacitors with improved cyclic stability from low-cost, abundant materials that have enhanced energy density. Supercapacitors are classified into two categories based on their charge storage mechanism: electrical double layer capacitors (EDLCs) and psuedocapacitors. In EDLCs, energy is stored on the interface of the electrode and electrolyte [1,2]. In the charging process of the EDLC, the electrons move from negative electrode (anode) to positive electrode (cathode) from the effect of external applied potential difference. Within the electrolyte, cations move towards anodes, and anions move towards cathodes. Electrons and ions move in opposite directions during the discharging process. In this type of supercapacitor, no redox reaction is involved, and it provides high power density, low energy density, and long cycle life. The most commonly used electrode materials in EDLCs are carbon-based, namely graphite, carbon nanotubes (CNTs), activated carbon, graphene, graphene oxide, reduced graphene oxide, etc. [3,4,5,6,7]. By contrast, in pseudo-type supercapacitors, chemical redox reactions are involved at the electrode/electrolyte interface giving rise to extra charge storage and resulting in higher energy density than the EDLC, but this may lead to lower cycling efficiency. Suitable materials to use as electrodes in this kind of supercapacitor require exhibiting a redox reaction. There is a large variety of electrode materials being proposed for pseudocapacitors such as organic materials (polyaniline and polypyrrole), metal oxides (NiO, Co_3_O_4_, CuO, NiCo_2_O_4_, etc.), metal sulfides (MoS_2_), etc. [8,9,10,11,12,13,14]. Carbon-based materials are highly abundant hence widely used in supercapacitors. Over the last many years, though, huge efforts have been made to produce carbon-based nanostructures for efficient performance in energy harvesting and storage devices, such as CNTs, graphene oxide, and graphene. Graphene oxide is an important derivative of graphite, which features carbon-oxygen (carboxylic or keto) and hydroxyl groups at its planes and edges. These functional groups make GO easier to handle/use than graphene in addition to being less costly to produce. Thanks to its functional groups, GO can interact with other molecular or ionic species, through, for example in electrostatic forces, hydrogen bonding, or covalent bonding. These oxygen-containing functional groups on its basal plane contribute additional pseudo capacitance and exhibit better capacitance [15,16,17]. However, the conductivity of the GO is lower than that of graphite or graphene, which hinders its performance in practical applications. Many efforts have been made to improve the conductivity of GO by making composites with conductive polymers, MOFs, transition metal oxides, etc. [18,19]. Originally high performance was achieved with rare metal oxides such as RuO_2_. The high cost of these metals makes their commercial use in pseudocapacitor devices prohibitive. Transition metal oxides have been investigated to replace these rare metals, with comparable performances. MnO_2_ is one of the best metal oxides being investigated as an electrode material for supercapacitors. Among the advantages of MnO_2_ are its tremendous capacitance, stability, high abundance, low cost, and non-toxicity [3,20]. There exists though a significant drawback of MnO_2_, which is its low conductivity [21]. Meanwhile, nanostructured NiO is strongly advocated for use in pseudocapacitors due to its stability and efficient redox reactions involved in energy storage. Zero-dimensional, one-dimensional, two-dimensional, and three-dimensional nanostructures of NiO have been proposed in different types of energy storage devices mostly in supercapacitors. All of these structures are synthesized by a number of different techniques, i.e., sol–gel, coprecipitation, hydrothermal, solvothermal, or electrodeposition. NiO is highly stable both thermally and chemically. Its electrochemical performance is influenced by the morphology and porosity of the material. The layered and porous layers have been proved attractive material features for supercapacitors [22,23]. Carbon-based materials and transition metal oxides have been used in oxygen evolution reactions (OERs), hydrogen evolution reactions (HERs), and oxygen reduction reactions (ORRs) solely and jointly many times. In the previous literature, the electrochemical performance of carbon-based materials have been significantly increased with the formation of their composite with other nanostructures [24,25,26,27,28].

In this work, we have focused on constructing a hybrid electrode structure for supercapacitor application taking advantage of GO as layered nanostructured template and NiO and MnO_2_ nanoparticles as synergistic pseudocapcitive oxides. The performance of MnO_2_/GO (MGO) and NiO/GO (NGO) was quite improved in comparison to the pristine GO. The ternary composite NiO/MnO_2_/GO (NMGO) exhibited a tremendously improved performance of 402 Fg^−1^ with an efficient cyclic stability. Motivated by the improved electrochemical performance of NMGO, a NMGO//MWCNT asymmetric device was assembled with NMGO and MWCNTs as positive and negative electrodes, respectively, which exhibited enhanced energy density, high power density, and excellent capacitance retention. Furthermore, our NMGO electrode exhibits higher oxygen evolution reaction activity with a lower onset potential of 1.592 V versus the reversible hydrogen electrode (RHE) at a current density of 10 mA·cm^−2^ rather than that of GO electrode (~1.701 V vs. RHE). The NMGO electrode also exhibited stability of electrochemical performance for 20 h by exhibiting constant current density close to the initial value determined by chronoamperometry. As a di-functional material, NMGO has broad application prospects.

## 2. Materials and Methods

### 2.1. Materials

All chemicals were used as analytical standards and used as received. Nickel chloride hexahydrate NiCl_2_ 6H_2_O (97% purity) was purchased from Riedel-deHaen, Seeize, Germany. Graphite Powder (research grade) Sodium hydroxide NaOH (98% purity) was purchased from DAEJUNG Company, Siheung-si, South-Korea, KMnO_4_, H_2_O_2_, and MnCl_2_ 4H_2_O was purchased from Scharlau, Barcelona, Spain. H_2_SO_4_, HCl, and H_3_PO_4_ was purchased from Merck, Rahway, New Jersy, United States. Absolute ethanol (99.8% purity), Isopropyl alcohol (99.5% purity), and Nefion@Perfluorinated resin solution were supplied by Sigma-Aldrich, Missouri, United States. Deionized (DI) water was purchased from VITRO Diagnostic Laboratories Islamabad, Pakistan.

### 2.2. Synthesis of GO

GO was synthesized by an improved method, which is also known as Tour’s method. In this process, concentrated 360 mL H_2_SO_4_ and 40 mL H3PO4 were mixed at a ratio of 9:1. The mixed solution was added slowly into a beaker containing a mixture of 3 g of graphite powder and 18 g of KMnO4. The entire mixture was then stirred continuously for 12 h at 50 °C. Then, the solution was cooled down to room temperature naturally. Subsequently, the mixture was poured onto the ice of 400 mL of deionized water along with 4 mL of 30% H_2_O_2_. Finally, the entire mixture was filtered and centrifuged. After centrifugation, the remaining solid material was washed repetitively with DI water and 30% HCl and ethanol, and the pH of the solution was adjusted to ~7. Finally, the solid material was freeze-dried to obtain graphene oxide.

### 2.3. Synthesis of NiO

NiO was synthesized by our previously reported co-precipitation method [29]. In this process, 12 g of nickel chloride was dissolved in 100 mL of DI water by magnetic stirring for 30 min. After complete dissolution of nickel chloride, a transparent, green solution appeared. In another beaker of 10 M solution of NaOH in 50 mL, DI water was made. Then, NaOH solution was added dropwise in nickel chloride solution while being vigorously stirred until the pH of the solution reached 10 and the solution became turbid green. Then, the precipitates were washed by centrifugation and vacuum filtration with ethanol and DI water sequentially three times to remove by-products. Then, the filtrates were collected and dried at 80 °C in an electric oven overnight. After complete drying, the powder was thermally annealed in a muffle furnace at 400 °C for 2 h with a ramp rate of 2 °C per minute. After annealing, the sample was powdered by mortar and pestle and stored in a vacuum desiccator.

### 2.4. Formation of NiO/MnO_2_/GO Composite

For NiO/MnO_2_/GO composite formation, firstly, GO was dispersed in DI water and sonicated for 1 h. Then, the optimized amount of NiO, MnO_2_ was added to the above solution. After several experiments, the ratio of NiO: MnO_2_:GO for composite formation was 1:1:4, 0:1:4, and 1:0:4 optimized by weight for NMGO, MGO, and NGO, respectively. The formed mixture was ultrasonicated for 2 h at room temperature. Then, the resultant precipitate was centrifuged with distilled water and ethanol and dried under vacuum.

### 2.5. Characterizations

Powder-based x-ray diffraction spectroscopy (XRD) was carried out for structural analysis of synthesized materials. The microstructural study was performed by scanning electron microscope (SEM) and atomic force microscope (AFM). Raman spectroscopy was carried out to study the vibrational modes of the material. All the electrochemical properties were studied by using the three-electrode system in a 1M Na_2_SO_4_ electrolyte. The active material on glassy carbon acted as a working electrode, while Pt mesh was used as a counter electrode, and Ag/AgCl was used as a reference electrode. The working electrode was made by drop casting a stable suspension of electrode material and Nafion solution in ethanol over a glassy carbon electrode (GCE) (diameter 3 mm) and drying it at 60 °C. The active mass 10 µg was deposited on GCE for testing. The capacitive performance of the material was examined by electrochemical techniques such as cyclic voltammetry (CV), galvanostatic charge and discharge (GCD), and electrochemical impedance spectroscopy (EIS). The specific capacitance of material was calculated by cyclic voltammetry using this formula:

(1) $$C_{sp}=\frac{\int I.v}{mu\Delta v}$$

Here ∫I.v is the area calculated from cyclic voltammogram, *m* is loading mass, *u* is scan rate, and ∆*v* is potential window.

The specific capacitance of material was calculated by galvanostatic charge/discharge using this formula [30]:

(2) $$C_{sp}=\frac{I\times \Delta t_{V_{o}-2V_{o}}}{V_{o}-V_{IR\;drop}}$$

The energy density of material was calculated by galvanostatic charge/discharge using this formula:

(3) $$E_{d}=\frac{1}{2}C_{sp}{\left(\Delta V\right)}^{2}$$

The power density of material was calculated by galvanostatic charge/discharge using this formula:

(4) $$P_{d}=\frac{E_{d}}{\Delta t_{V_{o}-2V_{o}}}$$

In these equations *I*, *V*, *t*, *C_sp_*, *E_d_*, and *P_d_* stand for electric current, potential voltage, discharging time, specific capacitance, energy density, and power density, respectively. Linear sweep and cyclic voltammetry (CV) were conducted at a scan rate of 10 mVs**^−^**^1^ without iR compensation in a 1.0 M KOH solution. The recorded electrode potential vs. Ag/AgCl was calculated and calibrated to reference hydrogen electrode (RHE) using the formula E_(RHE)_ = E_Ag/AgCl_ + 0.0592 pH + 0.196 [31,32]. The electrochemical active surface area (ECSA) was calculated from the double-layer specific capacitance by performing cyclic voltammetry (CV) at various scan rates of 5, 10, 15, 20, 25, 30, 35, 40, 45, and 50 mVs**^−^**^1^ in a potential window of 0−0.2 V vs. Ag/AgCl. Electrochemical impedance spectroscopy (EIS) was performed in the frequency range of (1 × 105) − 0.1 Hz at a potential of 0.5 V.

## 3. Results and Discussion

**Figure 1:**
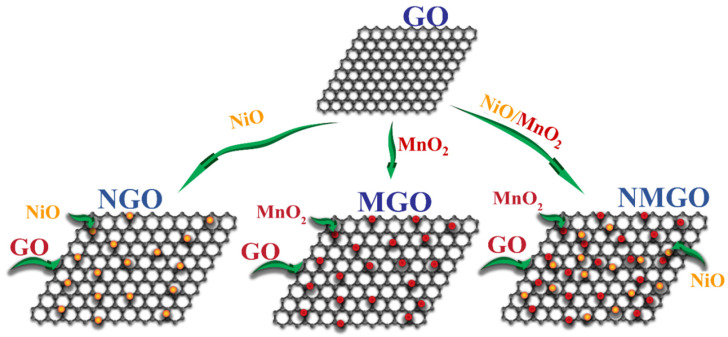
Schematic illustration for the formation of composites.

Two-dimensional GO sheets were decorated by MnO_2_ and NiO nanoparticles. A complete description of composite formation is explained in the experimental section and a schematic illustration for the composite formation is shown in Figure 1.

**Figure 2:**
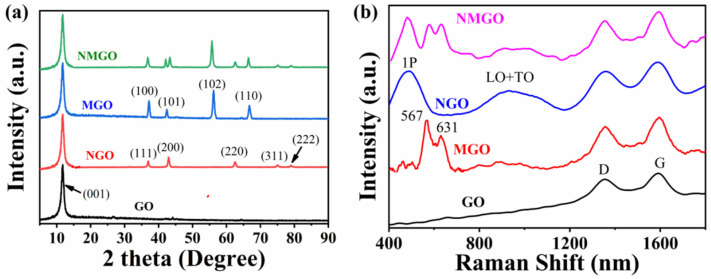
(**a**) XRD plot for GO, NGO, MGO, and NMGO and (**b**) RAMAN spectra for GO, NGO, MGO, and NMGO.

XRD analysis was carried out to investigate the formation of the materials. The XRD graph for the synthesized materials is shown in Figure 2a. Firstly, the XRD was performed for the GO, which exhibited a strong peak at 11°, which endorsed the successful synthesis of GO and was assigned to (001) plane [33]. No peak was observed around 24°, which affirmed that all the graphite powder was oxidized, and no impurities or unreacted graphite were present in GO. Then, we carried out XRD for the composites individually. The XRD spectra of NGO showed the peak of GO and other peaks were matched with JCPDF card number 01-071-1179, which showed the formation of the cubic structure of NiO. The peaks observed at 36.9°, 42.93°, 62.5°, 75.18°, and 78.9° were assigned to (111), (200), (220), (311), and (222), respectively. Then, the XRD for the MGO showed the presence of a GO peak, and additional peaks were matched with JCPDF card number 00-030-0820, which affirmed the formation of the hexagonal structure of MnO_2_. The peaks observed at 37.1°, 42.2°, 56.2°, and 66.7° were assigned to crystallite planes (100), (101), (002), and (110), respectively. The XRD graph for the NMGO showed the presence of GO, NiO, and MnO_2_, which endorsed the successful formation of NiO, MnO_2_, and GO composites. Furthermore, Raman spectroscopy was carried out to investigate the synthesized material. In Raman spectra for the GO, two distinct peaks were observed at 1352 cm^−1^ and 1587 cm^−1^, which were attributed to D and G bands. The D band was assigned to disordered structural defects, and the G band referred to the E_2g_ mode of Sp^2^ carbon [18,20,34]. In Raman spectra of MGO, two characteristics peaks of MnO_2_ were observed at 567 cm^−1^ and 631 cm^−1^ with the presence of D and G bands of GO. This endorsed the presence of GO and MnO_2_ in the composites [5,35,36,37]. In the case of Raman spectra for NGO, two significant peaks of GO representing D and G bands were assigned, and remaining broad peaks were observed at 488 cm^−1^and 929 cm^−1^ and assigned to first order (1P) phonon, longitudinal optical and transverse optical mode (LO + TO) in NiO [22,29,38,39,40,41]. In the case of Raman spectra for NMGO, the peaks of MnO_2_, NiO, and GO were observed, which endorsed the successful formation of an NiO/MnO_2_/GO composite as shown in Figure 2b.

**Figure 3:**
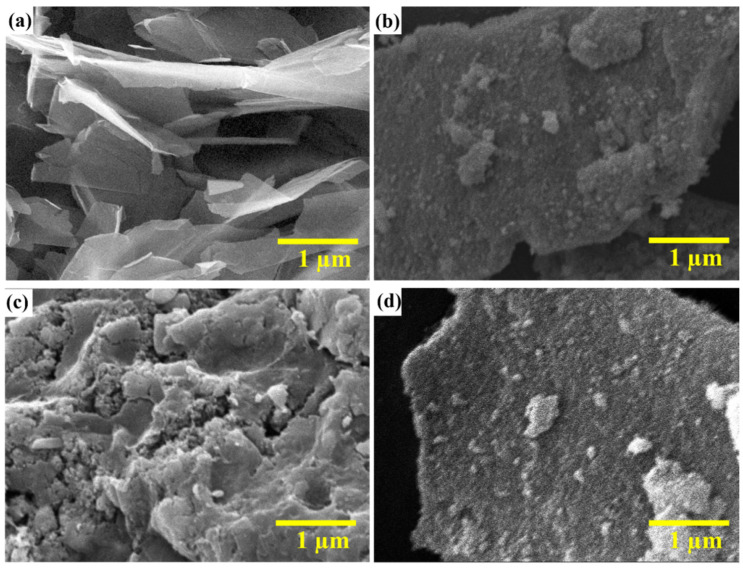
SEM image of (**a**) GO, (**b**) MGO, (**c**) NGO, and (**d**) NMGO.

SEM was performed to visualize the morphology of the synthesized materials. Figure 3a–d shows the SEM images for GO, MGO, NGO, and NMGO, respectively. Figure 3a exhibits the formation of 2D sheets with a thickness of less than 10 nm, which endorsed the formation of GO sheets. Then, Figure 3b–d shows that the GO sheets were covered with nanoparticles, which affirmed the successful addition of MnO_2_ and NiO nanoparticles on GO sheets. We have performed the particle size analyzer to visualize the size and particle-size distribution of the metal oxide nanoparticle. We observed that the average size for the NiO nanoparticle was 69 nm, and the size of most particles were between 50 and 70 nm. Furthermore, MnO_2_ nanoparticles were found more narrowly size distributed with an average particle size of 55 nm, and most particles were in the range of 40 to 60 nm as shown in Figure S3. AFM was also performed to further investigate the morphology of GO sheets. Figure S1a,b (supporting information) exhibits the formation of 2d sheets with a thickness of 8 nm, which affirmed the formation of GO sheets.

Firstly, cyclic voltammetry was carried out to study the electrochemical performance of the synthesized materials. Figure S2a–d in the supporting information shows the cyclic voltammogram for NMGO, NGO, MGO, and GO, respectively, at scan rates of 5 mVs^−1^, 10 mVs^−1^, 20 mVs^−1^, 40 mVs^−1^, 50 mVs^−1^, and 100 mVs^−1^. The cyclic voltammogram for the GO and MnO_2_ was close to the rectangular shape, which affirmed the EDLC type behaviour of the material. In NGO and NMGO, a minute contribution of pseudocapacitive behaviour was observed due to the presence of NiO in the composite. The area under the curve was improved by making a GO composite with metal oxide. A higher specific capacitance for NMGO was observed, which was due to the synergetic contribution of NiO and MnO_2_ with GO.

**Figure 4:**
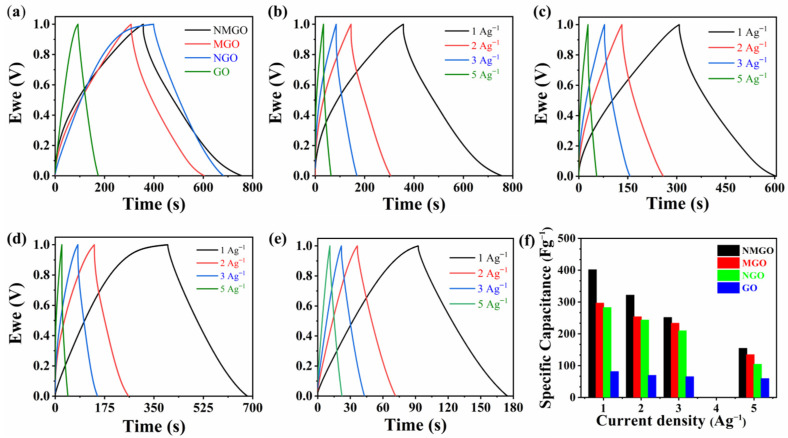
(**a**) Comparison of galvanostatic charge and discharge curve at the current density of 1 A·g^−1^ between NMGO, MGO, NGO, and GO; galvanostatic charge and discharge curve of (**b**) NMGO, (**c**) MGO, (**d**) NGO, and (**e**) GO; and (**f**) comparison of specific capacitance calculated by galvanostatic charge and discharge at different current densities between NMGO, MGO, NGO, and GO.

**Figure 5:**
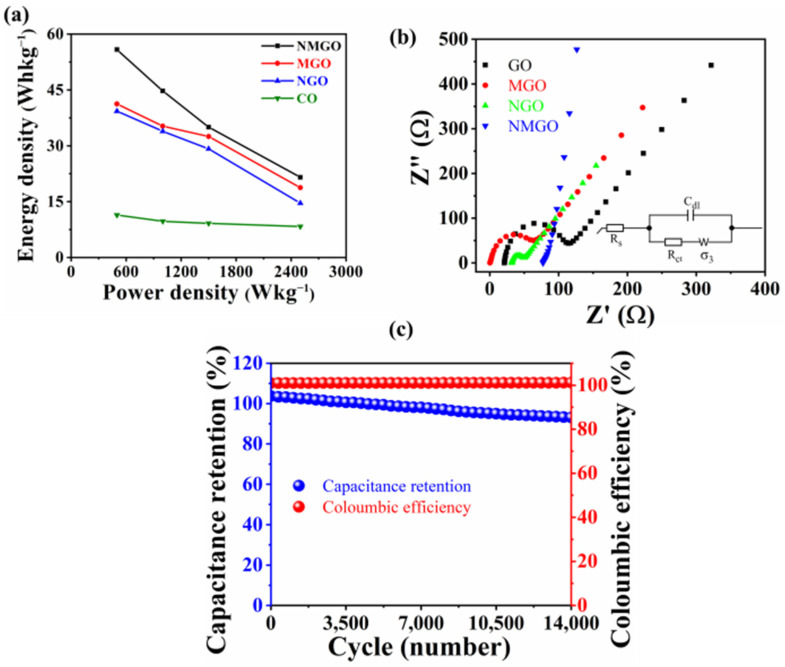
(**a**) Ragone plot for NMGO, MGO, NGO, and GO; (**b**) Nyquist plot for NMGO, MGO, NGO, and GO, and inset is the equivalent circuit used for z-fitting; (**c**) galvanostatic charge and discharge cyclic stability of NMGO.

Further GCD was carried out to study the electrochemical performance of the prepared composites. Figure 4a shows the comparison of the GCD graph for prepared materials at a current density of 1 A·g^−1^. The shape of the graph for GO and MGO was linear, which endorsed the EDLC-type capacitive behaviour, and the shape of the graph for NGO and NMGO was nonlinear, which endorsed the pseudocapacitive behaviour that was due to the presence of NiO. This information was in good agreement with CV curves. It was seen that the discharging time of GO was increased by making it composite with MnO_2_ and NiO, which revealed improvement in its performance. NMGO exhibited the specific capacitance of 402 F·g^−1^ at a current density of 1 A·g^−1^, which was quite higher than MGO (297 F·g^−1^), NGO (283 F·g^−1^), and GO (82 F·g^−1^). The remarkable performance of NMGO was achieved due to decoration of GO sheets with NiO and MnO_2_, which keeps sheets intercalated, provides additional paths in transportation of electrical charge, and reduces the charge transfer resistances. This improved the charge diffusion and the penetration of electrolytes in pores, which provides more space to store charge. Then, we performed the GCD for each material at different current densities to observe the detailed performance of the materials. Figure 4 b–e shows the GCD graph for NMGO, MGO, NGO, and GO at different current densities. It was seen that the specific capacitance for all the materials decreased with increasing current density. This phenomenon was caused due to the provision of less time for the electrolyte to penetrate the reactive sites. Figure 4f shows the comparison of specific capacitance for all the materials calculated by GCD at different current densities. Figure 5a shows the Ragone plot to visualize the relation between energy density and power density of the prepared material. These parameters have significant importance in practical application. Supercapacitors exhibit low energy density, which hinders its application in most of the practical applications. In this work, we have improved the energy density of the prepared materials. NMGO exhibited an energy density of 56 Wh^−1^ kg corresponding to the power density of 500 W kg^−1^, which is tremendously higher than MGO (41 Wh·kg^−1^), NGO (39 Wh·kg^−1^), and GO (11 Wh·kg^−1^).

EIS was carried out to study the detailed charge transfer mechanisms involved in the performance of prepared materials between electrode and electrolyte. Figure 5b shows the z-fitted Nyquist plot using the equivalent circuit shown inset to the Figure 5b for GO, NGO, MGO, and NMGO. The fitted graph consisted of two parts: one is a real axis and the second is a semicircle. The real axis of the graph exhibits the internal resistances of the system, i.e., resistance between the electrode material and electrolyte, electrolyte ionic resistance, electrode resistance, etc. [42]. The real axis is also used to study the diffusion coefficient of electrolyte with the electrode material. For ideal capacitive materials, the real axis should be vertical. The semicircle section exhibits the charge-transfer resistance of the electrode material. The diameter of the semicircle is dependent on charge-transfer resistance. The larger semicircle means a higher value of charge-transfer resistance. The semicircle for GO was higher as compared to other materials, which endorsed the higher amount of charge-transfer resistance [43]. The charge-transfer resistance of cobalt oxide is higher due to its randomly ordered morphology of nanowires. The pristine GO showed a high charge-transfer resistance up to 139 Ω, which hinders from being used as an electrode material in supercapacitors. The addition of metal oxides reduced the charge-transfer resistance due to their synergetic effect, large surface area, and creating a more reactive site to penetrate the electrolyte. NMGO showed a low charge transfer resistance of 34 Ω, which was lower than MGO (68 Ω), NGO (87 Ω), and GO. This reduction in charge transfer resistance affirmed the improvement in electrochemical properties.

Charge storage cyclic stability is the most important parameter for an electrode material to be used in practical application. We carried out the cyclic stability test for NMGO for 14,000 GCD cycles at a current density of 20 A·g^−1^ to visualize the stability of NMGO in sustaining a charge for a large number of cycles as shown in Figure 5c. It was observed that NMGO showed a coloumbic efficiency close to 100% for all cycles and a 93% capacitance retention after 14,000 GCD cycles. The performance was improved due to high surface area exhibited by ternary composite. We performed a BET analysis, which endorsed that the NMGO ternary composite and GO show specific surface areas of 102 m^2^ g^−1^ and 94 m^2^ g^−1^, respectively. The isotherm is shown in Figure S4. This loss in specific capacitance can be caused due to blocking the pores, which diminishes the electrode/electrolyte interface with the passage of charging/discharging cycles. Further, cyclic voltammetry at a slow scan rate was carried out after complete activation of material to visualize the electrochemical charge storage mechanism in detail as shown in Figure S5a. Firstly, observed anodic and cathodic peaks at a slow scan rate of CV curves were labeled, and the relation between peak current (*i*) and scan rate can be written as [44,45,46]:

*i = av^b^* (5)

Or

*log (i) = b log (v) + log (a)* (6)

Here, parameter *b* is important, as it endorsed the charge storage mechanism. Charge storage is controlled by the diffusion process; if its value is near 0.5, and if its value is near 1 or higher, it indicates that the charge storage is pseudocapacitive. The value of b was determined by slope of *log i* vs *log v* for specific peaks as shown in Figure S5b. The overall pseudocapacitive contribution is calculated by equation.

*i = k_1_v + k_2_v^1/2^* (7)

Here *k_1_v *and* k_2_v^1/2^* represent the pseudocapacitive and diffusion-econtrolled processes, respectively. The pseudocapacitive contributions are 29%, 34%, 39%, 46%, and 52% at scan rates of 0.1, 0.2, 0.3, 0.5, and 1 mVs^−1^, respectively, as shown in Figure S5c. 

A comparison in electrochemical performance of our prepared material is shown in Table S1, which affirmed the relatively improved performance of our material than those in the previous literature.

**Figure 6:**
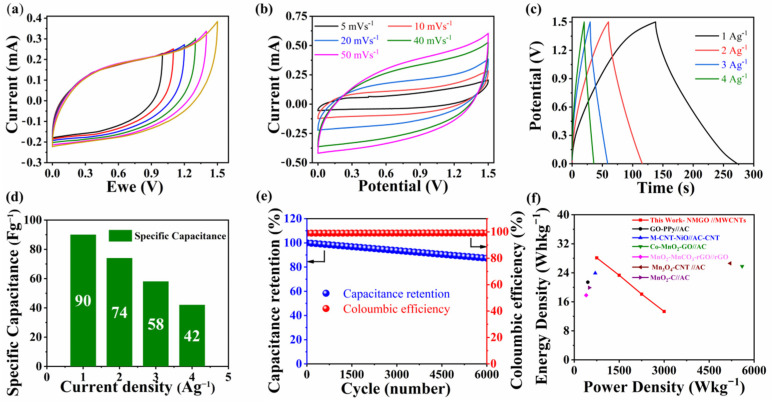
(**a**) CV curve of NMGO//MWCNT device at scan rate of 20 mVs^−1^ for different potential window; (**b**) cyclic voltammogram of the asymmetric device at different scan rates with fixed potential window; (**c**) GCD graph of asymmetric device at different current densities; (**d**) specific capacitance of NMGO//MWCNTs asymmetric device; (**e**) comparison between coloumbic efficiency and capacitance retention of asymmetric device; (**f**) Ragone plot of NMGO//MWCNT asymmetric device (this work), GO-PPy//AC [48], CNT-NiO//AC-CNT [47], Co-MnO_2_-GO//AC, MnO_2_-MnCO_3_-rGO//rGO [49], Mn_3_O_4_-CNT//AC [16], and MnO_2_-C//AC [50].

After achieving an improved electrochemical performance of NMGO, it was decided to fabricate an aqueous asymmetric supercapacitor using MWCNT as the negative electrode. The charge-to-mass ratio was calculated by $\frac{m_{+}}{m_{-}}=\frac{C_{-}\times \Delta V_{-}}{C_{+}\times \Delta V_{+}}\;$ [47]. Figure 6a shows the cyclic voltammogram of the NMGO//MWCNT asymmetric device with 1 M Na_2_SO_4_ electrolyte at a scan rate of 20 mVs^−1^. It can be seen that the fabricated asymmetric device shows CV curves close to the quasi-rectangular shape even for 1.5 V. Further, CV was carried out at different scan rates as shown in Figure 6b [48]. The CV analysis at different scan rates did not exhibit any significant distortions and indicated good rate capability of the asymmetric device. Further GCD was carried out to analyze the charge-storage capabilities of the device. The GCD curve for the assembled device is shown in Figure 6c. These curves exhibit a symmetric charge/discharge profile and endorse the ideal capacitive behaviour of assembled asymmetric device. The specific capacitances calculated from GCD curves were 90 F·g^−1^, 74 F·g^−1^, 58 F·g^−1^, and 42 F·g^−1^ at a current density of 1 A·g^−1^, 2 A·g^−1^, 3 A·g^−1^, and 4 A·g^−1^,respectively, as shown in Figure 6d. For practical application, cyclic stability is an important feature of the device. The cyclic stability of the NMGO//MWCNT device was examined by GCD cycles and exhibited the capacitance retention of 88% after 6000 GCD cycles as shown in Figure 6e. Furthermore, the energy density and power density were calculated using the following equations [8].

$$E=\frac{1}{2}\;C_{sp}\Delta V^{2}$$

$$P=\frac{E}{t}$$

Here *E* is energy density, *P* is power density achieved by the device based on active electrode materials, *C_sp_* is specific capacitance, Δ*V* is the operating potential window, and *t* is discharging time. The NMGO//MWCNT exhibited an excellent energy density of 28 Wh·kg^−1^ at the corresponding power density of 750 W kg^−1^. The power densities and corresponding energy densities of the NMGO//MWCNT device are summarized in Figure 6f.

**Figure 7:**
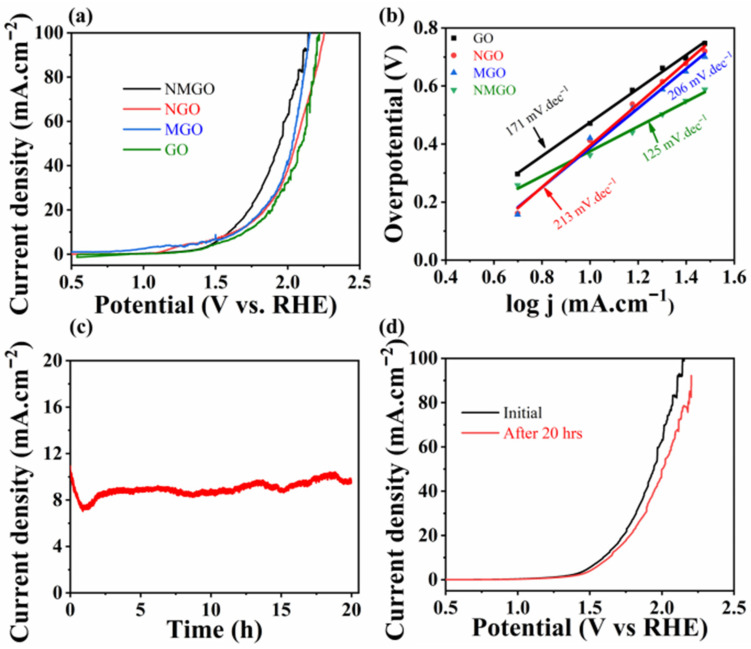
(**a**) OER polarization curves for prepared electrocatalyst in 1 M KOH, (**b**) Tafel slopes calculated from corresponding LSV curves, (**c**) chronoamperometric of NMGO for 20 h in 1 M KOH, (**d**) LSV curves of NMGO at 10 mVs^−1^ in 1 M KOH initially and after 20 h chronoamperometric test.

LSV was carried out to determine the electrocatalytic activity of prepared composites in 1 M KOH electrolyte. Figure 7a shows the polarization curves of pure GO and its composite with NiO and MnO_2_ with different ratios at the scan rate of 10 mVs^−1^ by using a three-electrode system. Here, all the potentials are measured with reference to a reversible hydrogen electrode (RHE). It was observed from the spectra that NMGO composites show OER activity greater than pure GO and its binary composites NGO and MGO owing to the large surface area and high electronic conductivity by the incorporation of GO nanosheets with NiO and MnO_2_ synergetically. The NMGO-composite electrode exhibits an onset potential of 1.592 V versus RHE at an anodic current density of 10 mA·cm^−2^, which is lower than that of GO (1.701 V vs. RHE), NGO (1.643 V vs. RHE), and MGO (1.651 V vs. RHE). The NMGO ternary composite showed a low overpotential 362 mV due to the synergetic presence of NiO and MnO_2_ with GO sheets. The introduction of these nanomaterials to GO sheets enhances the reactive sites and improves the electrochemical performance. The Tafel slope values of the prepared catalytic electrodes were also calculated from LSV data shown in Figure 7b. The Tafel slope of the NMGO electrode was 125 mV dec^−1^, which is lower than that of the MGO electrode (206 mV dec^−1^), NGO electrode (213 mV dec^−1^), and GO electrode (171 mV dec^−1^). To further investigate the stability of NMGO as an electrocatalyst, chronoamperometry was performed for 20 h as shown in Figure 7c. This affirmed the tremendous performance of NMGO with negligible loss in anodic current in alkaline medium. Afterward, a long-term stability test LSV was repeated to observe the performance of the catalyst after a 20 h chronoamperometry test as shown in Figure 7d.

## 4. Conclusions

An NiO/MnO_2_/GO composite was successfully synthesized by a wet chemical technique. The microstructural analysis of the composite showed the mesoporosity. The electronic conductivity of GO was improved by making its composite, which helped in the improvement of its electrochemical performance. The mesoporous structure of the composite allowed maximum interfacial contact between electrolyte and electrode to store more charges and enhanced the active sites’ contact with electrolytes to improve OER activity. The specific capacitance and charge stability of the ternary composite was improved by the synergetic effect of NiO, MnO_2_, and GO.

## Supplementary Materials

The following supporting information can be downloaded at: https://www.mdpi.com/article/10.3390/nano13010099/s1, Figure S1: AFM of NMGO, Figure S2 cyclic voltammogram of prepared materials, Figure S3: charge storage mechanism contribution, Figure S4: metal oxides particle size distribution, Figure S5: (a) Cyclic voltammogram at slow scn rates (b) *log i* vs. *log v* for NMGO composite, (c) Pseudo capacitive and diffusion controlled charge storage contribution at different scan rates; Table S1: Comparison of electrochemical performance of asymmetric supercapacitors assembled with different nanostructures.. References [3,48,51,52,53,54] are cited in the Supplementary Materials.

## References

[1] Abraham S., Prasankumar T., Vinoth Kumar K., Zh Karazhanov S., Jose S. (2020). Novel Lead Dioxide Intercalated Polypyrrole/Graphene Oxide Ternary Composite for High Throughput Supercapacitors. Mater. Lett..

[2] Hong J., Mengesha T.T., Hong S.-W., Kim H.-K., Hwang Y.-H. (2020). A Comparative Study of the Effects of Different Methods for Preparing Rgo/Metal-Oxide Nanocomposite Electrodes on Supercapacitor Performance. J. Kor. Phys. Soc..

[3] Miniach E., Śliwak A., Moyseowicz A., Fernández-Garcia L., González Z., Granda M., Menendez R., Gryglewicz G. (2017). MnO_2_/Thermally Reduced Graphene Oxide Composites for High-Voltage Asymmetric Supercapacitors. Electrochim. Acta.

[4] Tamilselvi R., Ramesh M., Lekshmi G.S., Bazaka O., Levchenko I., Bazaka K., Mandhakini M. (2020). Graphene Oxide – Based Supercapacitors from Agricultural Wastes: A Step to Mass Production of Highly Efficient Electrodes for Electrical Transportation Systems. Renew. Energy.

[5] Xia H., Wang Y., Lin J., Lu L. (2012). Hydrothermal Synthesis of MnO_2_/Cnt Nanocomposite with a Cnt Core/Porous MnO_2_ Sheath Hierarchy Architecture for Supercapacitors. Nanoscale Res. Lett..

[6] Miah M., Mondal T.K., Ghosh A., Saha S.K. (2020). Study of Highly Porous Zno Nanospheres Embedded Reduced Graphene Oxide for High Performance Supercapacitor Application. Electrochim. Acta.

[7] Zhao X., Liu X., Li F., Huang M. (2020). MnO_2_@ Nio Nanosheets@ Nanowires Hierarchical Structures with Enhanced Supercapacitive Properties. J. Mater. Sci..

[8] Ahsan M.T., Usman M., Ali Z., Javed S., Ali R., Farooq M.U., Akram M.A., Mahmood A. (2020). 3d Hierarchically Mesoporous Zinc-Nickel-Cobalt Ternary Oxide (Zn0.6ni0.8co1.6o4) Nanowires for High-Performance Asymmetric Supercapacitors. Front. Chem..

[9] Wang M., Yang J., Liu S., Li M., Hu C., Qiu J. (2020). Nitrogen-Doped Hierarchically Porous Carbon Nanosheets Derived from Polymer/Graphene Oxide Hydrogels for High-Performance Supercapacitors. J. Colloid Interface Sci..

[10] Chen I.W.P., Chou Y.-C., Wang P.-Y. (2019). Integration of Ultrathin Mos2/Pani/Cnt Composite Paper in Producing All-Solid-State Flexible Supercapacitors with Exceptional Volumetric Energy Density. J. Phys. Chem. C.

[11] Roy A., Ray A., Saha S., Ghosh M., Das T., Satpati B., Nandi M., Das S. (2018). Nio-Cnt Composite for High Performance Supercapacitor Electrode and Oxygen Evolution Reaction. Electrochim. Acta.

[12] Zhang Y., Shen Y., Xie X., Du W., Kang L., Wang Y., Sun X., Li Z., Wang B. (2020). One-Step Synthesis of the Reduced Graphene Oxide@Nio Composites for Supercapacitor Electrodes by Electrode-Assisted Plasma Electrolysis. Mater. Des..

[13] Usman M., Adnan M., Ahsan M.T., Javed S., Butt M.S., Akram M.A. (2021). In Situ Synthesis of a Polyaniline/ Fe-Ni Codoped Co3o4 Composite for the Electrode Material of Supercapacitors with Improved Cyclic Stability. ACS Omega.

[14] Muhammad A., Muhammad U., Muhammad Aftab A., Sofia J., Saqib A., Iftikhar A., Mohammad I. (2021). Study of Magnetic and Dielectric Properties of Znfe2o4/Cocr2o4 Nanocomposites Produced Using Sol-Gel and Hydrothermal Processes. J. Alloys Comp..

[15] Zhou X., Meng T., Yi F., Shu D., Han D., Zhu Z., Gao A., Liu C., Li X., Yang K. (2020). Supramolecular-Induced Confining Methylene Blue in Three-Dimensional Reduced Graphene Oxide for High-Performance Supercapacitors. J. Power Sources.

[16] Cheng H., Duong H.M., Jewell D. (2016). Three Dimensional Manganese Oxide on Carbon Nanotube Hydrogels for Asymmetric Supercapacitors. RSC Adv..

[17] Hwang S.-G., Hong J.-E., Kim G.-O., Jeong H.M., Ryu K.-S. (2012). Graphene Anchored with Nio-MnO_2_ Nanocomposites for Use as an Electrode Material in Supercapacitors. ECS Sol. State Lett..

[18] Seredych M., Bandosz T.J. (2012). Evaluation of Go/MnO_2_ Composites as Supercapacitors in Neutral Electrolytes: Role of Graphite Oxide Oxidation Level. J. Mater. Chem..

[19] Zhang L., Tian Y., Song C., Qiu H., Xue H. (2020). Study on Preparation and Performance of Flexible All-Solid-State Supercapacitor Based on Nitrogen-Doped Rgo/Cnt/MnO_2_ Composite Fibers. J. Alloys Comp..

[20] Zhao Z., Shen T., Liu Z., Zhong Q., Qin Y. (2020). Facile Fabrication of Binder-Free Reduced Graphene Oxide/MnO_2_/Ni Foam Hybrid Electrode for High-Performance Supercapacitors. J. Alloys Comp..

[21] Huang M., Zhang Y., Li F., Zhang L., Wen Z., Liu Q. (2014). Facile Synthesis of Hierarchical Co3o4@MnO_2_ Core–Shell Arrays on Ni Foam for Asymmetric Supercapacitors. J. Power Sources.

[22] Qiu Z., He D., Wang Y., Zhao X., Zhao W., Wu H. (2017). High Performance Asymmetric Supercapacitors with Ultrahigh Energy Density Based on Hierarchical Carbon Nanotubes@Nio Core–Shell Nanosheets and Defect-Introduced Graphene Sheets with Hole Structure. RSC Adv..

[23] Kate R.S., Khalate S.A., Deokate R.J. (2018). Overview of Nanostructured Metal Oxides and Pure Nickel Oxide (Nio) Electrodes for Supercapacitors: A Review. J. Alloys Comp..

[24] Zhang R., Zhang Y.-C., Pan L., Shen G.-Q., Mahmood N., Ma Y.-H., Shi Y., Jia W., Wang L., Zhang X. (2018). Engineering Cobalt Defects in Cobalt Oxide for Highly Efficient Electrocatalytic Oxygen Evolution. ACS Catal..

[25] Aslam S., Sagar R.U.R., Kumar H., Zhang G., Nosheen F., Namvari M., Mahmood N., Zhang M., Qiu Y. (2020). Mixed-Dimensional Heterostructures of Hydrophobic/Hydrophilic Graphene Foam for Tunable Hydrogen Evolution Reaction. Chemosphere.

[26] Tahir M., Cao C., Mahmood N., Butt F.K., Mahmood A., Idrees F., Hussain S., Tanveer M., Ali Z., Aslam I. (2014). Multifunctional G-C(3)N(4) Nanofibers: A Template-Free Fabrication and Enhanced Optical, Electrochemical, and Photocatalyst Properties. ACS Appl. Mater. Interfaces.

[27] Babar N.U.A., Joya K.S., Ehsan M.A., Khan M., Sharif M. (2019). Noble-Metal-Free Colloidal-Copper Based Low Overpotential Water Oxidation Electrocatalyst. ChemCatChem.

[28] Narwade S.S., Mali S.M., Digraskar R.V., Sapner V.S., Sathe B.R. (2019). Ni/Nio@Rgo as an Efficient Bifunctional Electrocatalyst for Enhanced Overall Water Splitting Reactions. Int. J. Hydrogen Energy.

[29] Usman M., Adnan M., Ali S., Javed S., Akram M.A. (2020). Preparation and Characterization of Pani@Nio Visible Light Photocatalyst for Wastewater Treatment. Chem. Sel..

[30] Zhang S., Pan N. (2015). Supercapacitors Performance Evaluation. Adv. Energy Mater..

[31] Abid A.G., Manzoor S., Usman M., Munawar T., Nisa M.U., Iqbal F., Ashiq M.N., Najam-ul-Haq M., Shah A., Imran M. (2021). Scalable Synthesis of Sm2o3/Fe2o3 Hierarchical Oxygen Vacancy-Based Gyroid-Inspired Morphology: With Enhanced Electrocatalytic Activity for Oxygen Evolution Performance. Energy Fuels.

[32] Sadaqat M., Manzoor S., Aman S., Gouadria S., Usman M., Joya K.S., Najam-Ul-Haq M., Hassan H.M.A., Ashiq M.N., Taha T. (2022). Mn-Based Hierarchical Polyhedral 2d/3d Nanostructures Mnx2 (X= S, Se, Te) Derived from Mn-Based Metal–Organic Frameworks as High-Performance Electrocatalysts for the Oxygen Evolution Reaction. Energy Fuels.

[33] Wang H., Fu Q., Pan C. (2019). Green Mass Synthesis of Graphene Oxide and Its MnO_2_ Composite for High Performance Supercapacitor. Electrochim. Acta.

[34] Kim H.J., Lee S.-Y., Sinh L.H., Yeo C.S., Son Y.R., Cho K.R., Song Y., Ju S., Shin M.K., Park S.-J. (2017). Maximizing Volumetric Energy Density of All-Graphene-Oxide-Supercapacitors and Their Potential Applications for Energy Harvest. J. Power Sources.

[35] Xiong S., Zhang X., Chu J., Wang X., Zhang R., Gong M., Wu B. (2018). Hydrothermal Synthesis of Porous Sugarcane Bagasse Carbon/MnO_2_ Nanocomposite for Supercapacitor Application. J. Elect. Mater..

[36] Han X., Cheng F., Chen C., Hu Y., Chen J. (2014). Uniform MnO_2_ Nanostructures Supported on Hierarchically Porous Carbon as Efficient Electrocatalysts for Rechargeable Li-O_2_ Batteries. Nano Res..

[37] Freitas R.M., Perilli T.A.G., Ladeira A.C.Q. (2013). Oxidative Precipitation of Manganese from Acid Mine Drainage by Potassium Permanganate. J. Chem..

[38] Liu C., Li C., Ahmed K., Mutlu Z., Ozkan C.S., Ozkan M. (2016). Template Free and Binderless Nio Nanowire Foam for Li-Ion Battery Anodes with Long Cycle Life and Ultrahigh Rate Capability. Sci. Rep..

[39] Mironova-Ulmane N., Kuzmin A., Sildos I., Puust L., Grabis J. (2019). Magnon and Phonon Excitations in Nanosized Nio. Latv. J. Phys. Tech. Sci.

[40] Thema F.T., Manikandan E., Gurib-Fakim A., Maaza M. (2016). Single Phase Bunsenite Nio Nanoparticles Green Synthesis by Agathosma Betulina Natural Extract. J. Alloys Comp..

[41] Tian Y., Gong L., Qi X., Yang Y., Zhao X. (2019). Effect of Substrate Temperature on the Optical and Electrical Properties of Nitrogen-Doped Nio Thin Films. Coatings.

[42] Ramadan A., Anas M., Ebrahim S., Soliman M., Abou-Aly A. (2020). Effect of Co-Doped Graphene Quantum Dots to Polyaniline Ratio on Performance of Supercapacitor. J. Mater. Sci. Mater. Electr..

[43] Ramadan A., Anas M., Ebrahim S., Soliman M., Abou-Aly A.I. (2020). Polyaniline/Fullerene Derivative Nanocomposite for Highly Efficient Supercapacitor Electrode. Int. J. Hydrogen Energy.

[44] Usman M., Ahsan M.T., Javed S., Ali Z., Zhan Y., Ahmed I., Butt S., Islam M., Mahmood A., Akram M.A. (2022). Facile Synthesis of Iron Nickel Cobalt Ternary Oxide (Fnco) Mesoporous Nanowires as Electrode Material for Supercapacitor Application. J. Mater..

[45] Zhang K., Park M., Zhou L., Lee G.-H., Li W., Kang Y.-M., Chen J. (2016). Urchin-Like Cose2 as a High-Performance Anode Material for Sodium-Ion Batteries. Adv. Funct. Mater..

[46] Ali Z., Tang T., Huang X., Wang Y., Asif M., Hou Y. (2018). Cobalt Selenide Decorated Carbon Spheres for Excellent Cycling Performance of Sodium Ion Batteries. Energy Storage Mater..

[47] Lai Y.-H., Gupta S., Hsiao C.-H., Lee C.-Y., Tai N.-H. (2020). Multilayered Nickel Oxide/Carbon Nanotube Composite Paper Electrodes for Asymmetric Supercapacitors. Electrochim. Acta.

[48] Fan L.-Q., Liu G.-J., Wu J.-H., Liu L., Lin J.-M., Wei Y.-L. (2014). Asymmetric Supercapacitor Based on Graphene Oxide/Polypyrrole Composite and Activated Carbon Electrodes. Electrochim. Acta.

[49] Liu Y., He D., Wu H., Duan J., Zhang Y. (2015). Hydrothermal Self-Assembly of Manganese Dioxide/Manganese Carbonate/Reduced Graphene Oxide Aerogel for Asymmetric Supercapacitors. Electrochim. Acta.

[50] Wang G., Xu H., Lu L., Zhao H. (2015). One-Step Synthesis of Mesoporous MnO_2_/Carbon Sphere Composites for Asymmetric Electrochemical Capacitors. J. Mater. Chem. A.

[51] Chai Y., Li Z., Wang J., Mo Z., Yang S. (2019). Construction of hierarchical holey graphene/MnO_2_ composites as potential electrode materials for supercapacitors. J. Alloys Compd..

[52] Qiu S., Li R., Huang Z., Huang Z., Tsui C.P., He C., Han X., Yang Y. (2019). Scalable sonochemical synthesis of petal-like MnO_2_/graphene hierarchical composites for high-performance supercapacitors. Compos. Part B Eng..

[53] Chen Y., Zhang J., Li M., Yang C., Zhang L., Wang C., Lu H. (2018). Strong interface coupling and few-crystalline MnO_2_/Reduced graphene oxide composites for supercapacitors with high cycle stability. Electrochim. Acta.

[54] Hao H., Wang J., Lv Q., Jiao Y., Li J., Li W., Akpinar I., Shen W., He G. (2020). Interfacial engineering of reduced graphene oxide for high-performance supercapacitor materials. J. Electroanal. Chem..

