# Supplementary material for: Nanoengineering of NiO/MnO2/GO Ternary Composite for Use in High-Energy Storage Asymmetric Supercapacitor and Oxygen Evolution Reaction (OER)
Source: Nanomaterials (Basel). 2022 Dec 25;13(1):99. doi: 10.3390/nano13010099 (PMC9823737; doi:10.3390/nano13010099)
Supplement: Supplementary file 1 [file nanomaterials-13-00099-s001.zip › nanomaterials-2006432-supplementary.pdf]

Supporting Information

Nanoengineering of NiO/MnO_2/GO Ternary Composite for Use in High-Energy Storage Asymmetric Supercapacitor and Oxygen Evolution Reaction (OER)

Natasha Arshad [1,2], Muhammad Usman [2], Muhammad Adnan [2], Muhammad Tayyab Ahsan [3], Mah Rukh Rehman [2], Sofia Javed [2], Zeeshan Ali [2], M. Aftab Akram [4,*], George P Demopoulos [5] and Asif Mahmood [6,7,*]

[1] Department of Physics, Government College Women University, Sialkot 51310, Pakistan
[2] School of Chemical and Materials Engineering, National University of Sciences and Technology (NUST), Sector H-12, Islamabad 44000, Pakistan
[3] School of Materials Science and Engineering, Peking University, Beijing 100871, China
[4] Department of Materials Science & Engineering, Pak-Austria Fachhochschule, Institute of Applied Sciences & Technology, Khanpur Road, Mang, Haripur 22650, Pakistan
[5] Materials Engineering, McGill University, Montreal, QC H3A 0C5, Canada
[6] Center for Clean Energy Technology, School of Mathematical and Physical Science, Faculty of Science, University of Technology Sydney, Sydney 2007, Australia
[7] School of Chemical and Biomoecular Engineering, Faculty of Engineering, The University of Sydney, Sydney 2006, Australia
* Correspondence: aftab.akram@fcm3.paf-iast.edu.pk or aftabi101@gmail.com (M.A.A.); asif.mahmood@uts.edu.au or asif.mahmood@sydney.edu.au (A.M.)

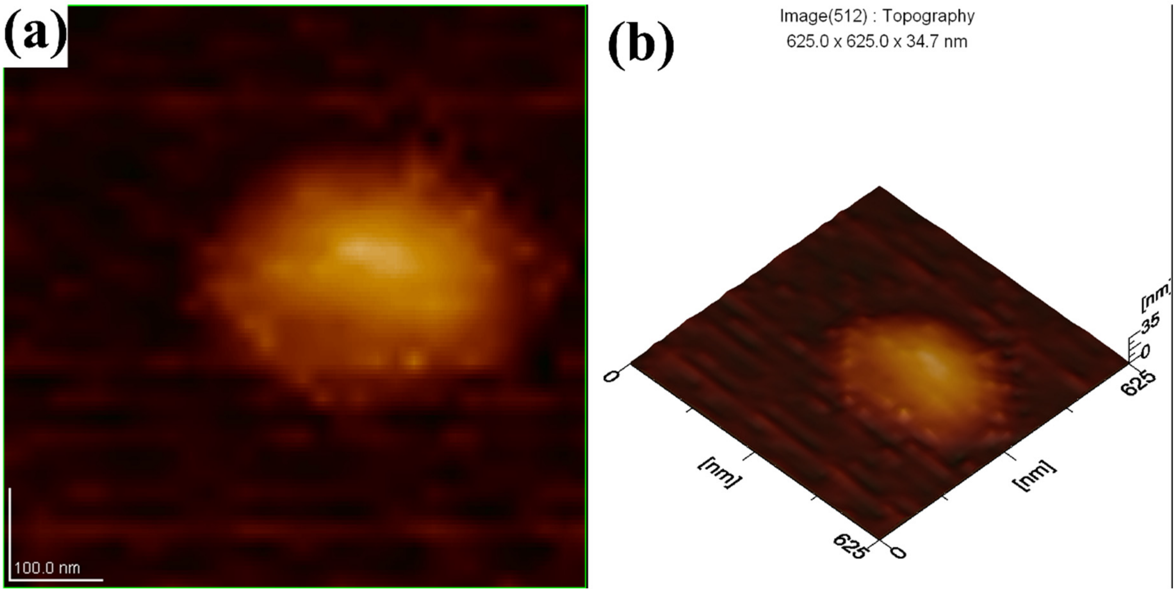

Figure S1. **(a)** AFM scanned 2D image of GO and **(b)** AFM scanned 3D image of GO.

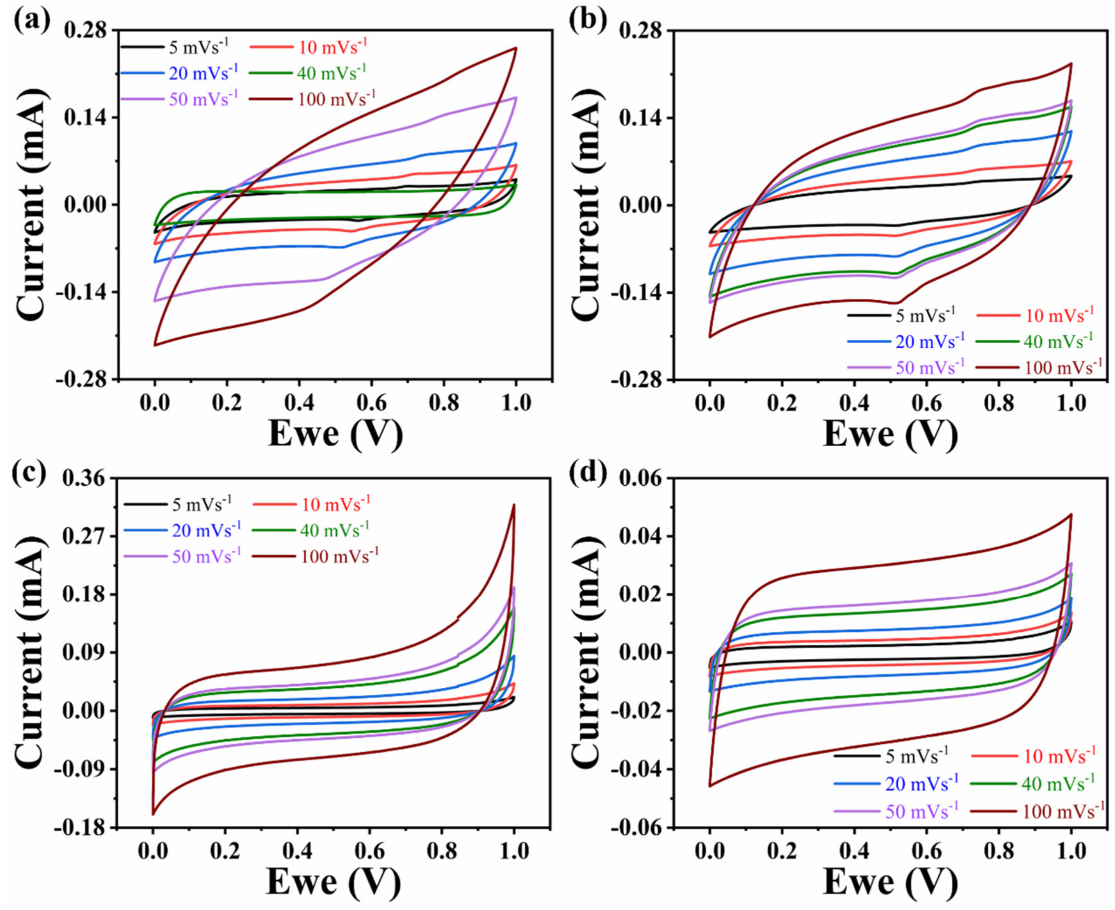

Figure S2. Cyclic Voltammogram at different scan rate for **(a)** NMGO, **(b)** NGO, **(c)** MGO and **(d)** GO.

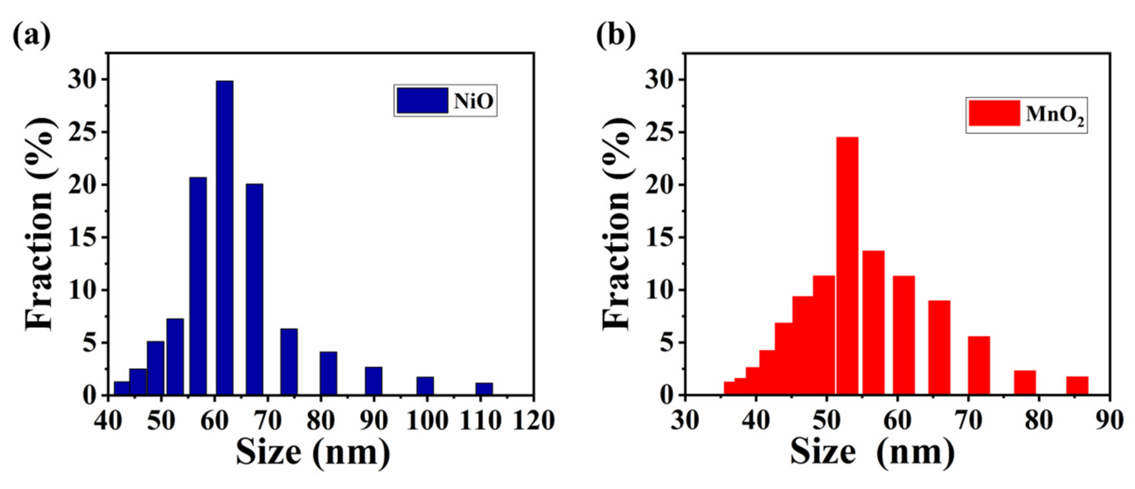

Figure S3. Particle size distribution of (a) NiO and (b) MnO_2.

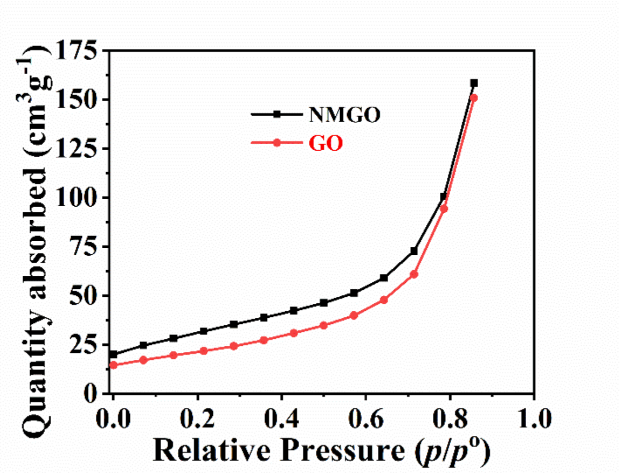

Figure S4. N_2 adsorption isotherm at 77K of NMGO composite.

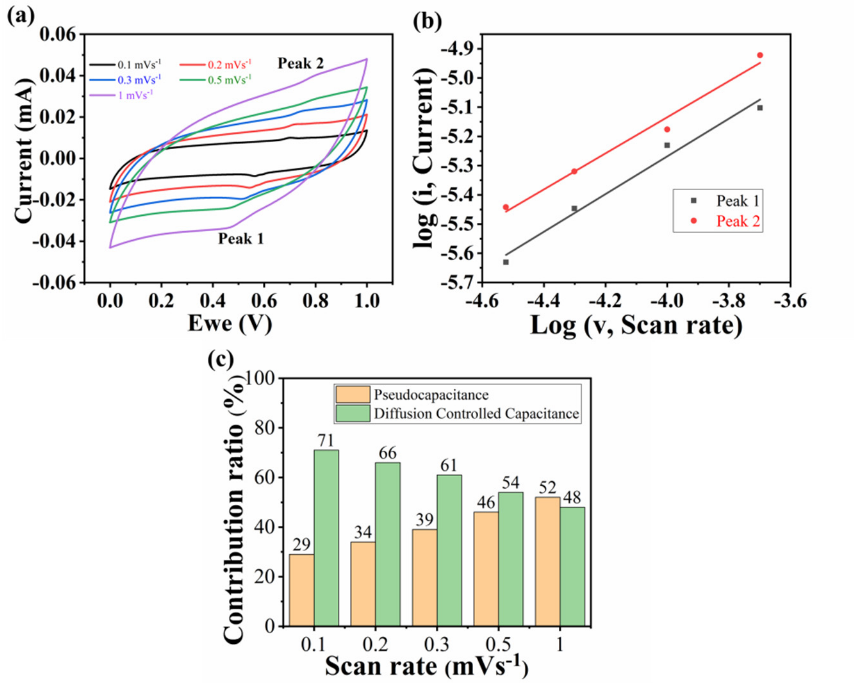

Figure S5. (a) Cyclic voltammogram at slow scn rates (b) *log i* vs *log v* for NMGO composite, (c) Pseudo capacitive and diffusion controlled charge storage contribution at different scan rates.

Table S1. Comparison of electrochemical performance of asymmetric supercapacitors assembled with different nanostructures.

Electrode material	Synthesis process	Specific Capacitance	Operating Potential Window (V)	Current Density	Capacitance retention	Ref
**graphene/MnO_2**	Hydrothermal	192 .2Fg^{-1}	-0.2-0.7	0.5 Ag^{-1}	79 % after 5000 cycles	[1]
**graphene/MnO_2**	Sonochemical	292 Fg^{-1}	-0.2-0.8	5 mV/s^{-1}	91 % after 1000 cycles	[2]
**rGO/MnO_2**	Reflux	234 Fg^{-1}	0-0.8	0.1 Ag^{-1}	100 % after 20000 cycles	[3]
**RGO quantam dots**	hydrothermal	312 Fg^{-1}	-0.2-0.8	0.5 Ag^{-1}	91 % after 10000 cycles	[4]

**GO/Polypyrole**	In situ oxidation polymerization	323 Fg^{-1}	0-0.8	0.25 Ag^{-1}	78 % after 5000 cycles	[5]
**MnO_2/rGO**	Hydrothermal	122 Fg^{-1}	-0.1-0.7	0.2 Ag^{-1}	92 % after 5000 cycles	[6]
**GO/MnO_2/NiO**	Hydrothermal	402 Fg^{-1}	0-1	1 Ag^{-1}	93% after 14000 cycles	This work

References:

1. Chai, Y., et al., *Construction of hierarchical holey graphene/MnO2 composites as potential electrode materials for supercapacitors.* Journal of Alloys and Compounds, 2019. **775**: p. 1206-1212.
2. Qiu, S., et al., *Scalable sonochemical synthesis of petal-like MnO2/graphene hierarchical composites for high-performance supercapacitors.* Composites Part B: Engineering, 2019. **161**: p. 37-43.
3. Chen, Y., et al., *Strong interface coupling and few-crystalline MnO2/Reduced graphene oxide composites for supercapacitors with high cycle stability.* Electrochimica Acta, 2018. **292**: p. 115-124.
4. Hao, H., et al., *Interfacial engineering of reduced graphene oxide for high-performance supercapacitor materials.* Journal of Electroanalytical Chemistry, 2020. **878**.
5. Fan, L.-Q., et al., *Asymmetric supercapacitor based on graphene oxide/polypyrrole composite and activated carbon electrodes.* Electrochimica Acta, 2014. **137**: p. 26-33.
6. Miniach, E., et al., *MnO2/thermally reduced graphene oxide composites for high-voltage asymmetric supercapacitors.* Electrochimica Acta, 2017. **240**: p. 53-62.
